# Advances in the comprehensive mechanisms, diagnosis, and treatment of heatstroke-induced coagulopathy

**DOI:** 10.3389/fcell.2025.1596039

**Published:** 2025-07-15

**Authors:** Chuhan Xiang, Ling Gao, Xuhui Liu, Jun Xiang, Bin Zhu, Shihui Fu, Jianhui Zhao, Qing Song

**Affiliations:** ^1^ Department of Cardiology, The 71st Group Army Hospital of People’s Liberation Army, Xuzhou, Jiangsu, China; ^2^ Department of Cardiology, The Affiliated Huaihai Hospital of Xuzhou Medical University, Xuzhou, Jiangsu, China; ^3^ Chinese People’s Liberation Army Heatstroke Prevention and Treatment Research Center, Sanya, Hainan, China; ^4^ Department of Pharmacy, The 71st Group Army Hospital of People’s Liberation Army, Xuzhou, Jiangsu, China; ^5^ Department of Pharmacy, The Affiliated Huaihai Hospital of Xuzhou Medical University, Xuzhou, Jiangsu, China; ^6^ Neurology Department, The Second Hospital of Lanzhou University, Lanzhou, China; ^7^ Department of Cardiology, Hainan Hospital of Chinese People’s Liberation Army General Hospital, Hainan Geriatric Disease Clinical Medical Research Center, Hainan Branch of China Geriatric Disease Clinical Research Center, Sanya, China; ^8^ Department of Geriatric Cardiology, Chinese People’s Liberation Army General Hospital, Beijing, China; ^9^ Department of Critical Care Medicine, Hainan Hospital of Chinese People’s Liberation Army People’s Liberation Army General Hospital, Sanya, Hainan, China

**Keywords:** heatstroke, heatstroke-induced coagulopathy, diagnostic criteria, pathogenesis, substitute, anticoagulation

## Abstract

Heatstroke (HS) is a life-threatening condition that is precipitated by heat stress. The coagulation disorder that consequentially develops is termed heatstroke-induced coagulopathy (HIC). HIC typically presents as either hypocoagulopathy or hypercoagulopathy and is a principal complication that contributes to HS-associated mortality. There is no current consensus regarding HIC pathophysiology and specific pharmacological treatments. We review the diagnostic approaches, underlying mechanisms, and therapeutic interventions for HIC, with a particular emphasis on pharmacological treatments that include replacement therapy, anticoagulant therapy, traditional Chinese medicine, and novel therapies. The aim of this study is to enhance HIC awareness among clinicians and researchers and discuss the latest treatment strategy advancements to ultimately facilitate comprehensive HIC management and reduce its mortality rate.

## 1 Introduction

Heatstroke (HS) is a severe heat-related condition that results from a thermoregulatory function imbalance induced by heat stress. It leads to excessive heat production and insufficient heat dissipation (Epstein and Yanovich, 2019). HS has a rapid onset and progression and is associated with a high mortality rate and significant morbidity. The HS incidence has been increasing annually with ongoing global climate warming. The number of pyrexia visits in China in 2022 was 539,000, a figure that represents a 180.77% increase from 2021 (Song et al., 2025). In 2023, the global cases of heat-related illnesses further increased, with 119,605 related visits to Emergency Departments the United States (Khan and Mubeen, 2025).Furthermore, a big data analysis has predicted that the HS incidence will escalate by at least sixfold by 2100. This will pose significant challenges for healthcare workers (Iba et al., 2023a; Han et al., 2022). HS-induced coagulation dysfunction is referred to as heatstroke-induced coagulopathy (HIC) (Iba et al., 2023a), and it is one of the most severe HS complications. HIC often presents as hypocoagulopathy and/or hypercoagulopathy and is clinically characterized by widespread microcirculatory thrombosis accompanied by varying degrees of bleeding events. It can lead to circulatory failure and ultimately death. The HIC incidence is high, with occurrences of disseminated intravascular coagulation (DIC) ranging from approximately 11%–48% (Iba et al., 2022; Misset et al., 2006; Hifumi et al., 2018). HIC is significantly positively correlated with multiple organ dysfunction syndrome in HS patients (Liu et al., 2020). The mortality rate significantly increases once patients progress to the DIC stage, the most severe state of HIC coagulation dysfunction (Hifumi et al., 2018; Zhong et al., 2022). HIC treatment typically consists of traditional therapies for coagulation dysfunction. In this study, we comprehensively review the comprehensive mechanisms, diagnostic criteria, and pharmacological treatments for HIC, including alternative therapies, anticoagulant treatments, herbal remedies, and emerging treatment modalities. The aim of this review is to provide foundations for the development of effective therapeutic strategies.

## 2 HIC diagnostic criteria

The diagnostic guidelines of the Japanese Association for Acute Medicine (JAAM) emphasize that the coagulation dysfunction is a critical diagnostic HS criterion. In 2019, Ohbe et al. (2019) introduced the concept of HIC. HIC diagnosis previously relied on the diagnostic criteria for septic DIC established by the International Society on Thrombosis and Haemostasis (ISTH) (Zhang et al., 2019). Subsequently, in 2023, an expert group from the Chinese military specializing in HS prevention and treatment presented the first global expert consensus on HIC titled the “China Expert Consensus on Diagnosis and Treatment of Heatstroke-Induced Coagulopathy”. This document draws on extensive clinical experience and research findings and provides a clear definition of HIC. It also introduces a diagnostic scoring system for its initial emergency screening (Liu et al., 2020). Prior to this, the diagnostic DIC criteria associated with HS (HS-related DIC) were aligned with those for septic DIC (Table 1) (Song et al., 2023; Lin et al., 2023).

**TABLE 1:** HIC and ISTH-DIC diagnostic criteria.

Items	HIC diagnostic criteria	ISTH-DIC diagnostic criteria
Maximum core temp (°C)	<40; 0 40∼41.9; 1 ≥42; 2	—
D-dimer (µg/mL)	<1; 0 1∼2.4; 1 ≥2.5; 2	<2.5; 0 2.5∼4.9; 2 ≥5; 3
Prothrombin time (s)	<2; 0 2∼<3.9; 1 ≥4; 2	<3; 0 3∼5.9; 1 ≥6; 2
Platelet count (×10^9^/L)	—	≥100; 0 50∼99; 1 <50; 2
Fibrinogen (g/L)	—	≥1.0; 0 <1.0; 1
Diagnosis	≥3	≥5

HIC: heatstroke-induced coagulopathy; ISTH: International Society on Thrombosis and Haemostasis; DIC: disseminated intravascular coagulation.

## 3 Mechanisms of HIC occurrence

### 3.1 Heat stress inducing HIC

**FIGURE 1:**
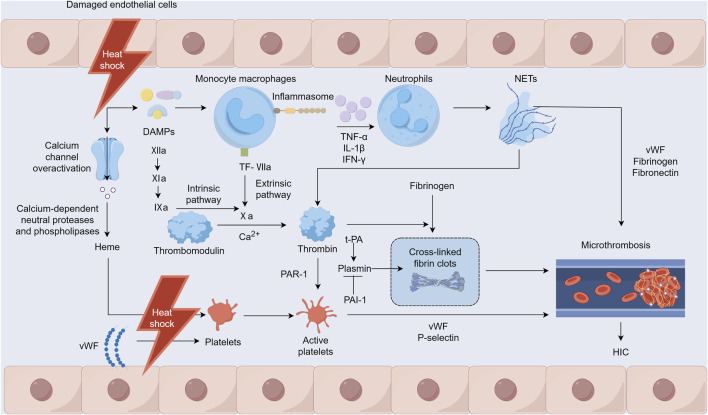
Mechanisms of heatstroke-induced coagulopathy occurrence. Notes: DAMPs: damage-associated molecular patterns, NETs: Neutrophil extracellular traps, TF: tissue factor, PAR-1: protease-activated receptor-1, t-PA: tissue-type plasminogen activator, PAI-1: plasminogen activator inhibitor-1, vWF: von Willebrand factor, HIC: heatstroke-induced coagulopathy.

Heat stress can directly or indirectly damage various cells, including monocytes, neutrophils, platelets, and endothelial cells, with vascular endothelial cells being the primary target of heat stress injuries (Figure 1) (Umemura et al., 2018). Heat stress may also directly or indirectly activate platelets through heme (Iba et al., 2023a). The adenosine diphosphate-induced platelet aggregation function is enhanced, while endothelial cells damaged by heat stress release von Willebrand factor (vWF). This factor increases platelet adhesion and aggregation in the microcirculation and leads to extensive microvascular thrombosis (Iba et al., 2023b). Heat stress disrupts cytoskeletal proteins and alters platelet morphology and significantly inhibits platelet aggregation when the core temperature rises to ≥43°C, which in turn impairs hemostatic function (Iba et al., 2023c; Majeed, 2009). In addition, during heat stress, excessive activation of calcium channels triggers calcium-dependent neutral proteases and phospholipases that damage myofibrils, the cytoskeleton, and membrane proteins. This damage results in the leakage of substances such as myoglobin and heme, thereby causing kidney function damage (Yamazawa et al., 2021). iHeat stress can also directly affect coagulation function and fibrinolysis, worsening cell damage and leading to further deterioration of coagulation dysfunction associated with HS.

### 3.2 HIC inflammation-coagulation interactions

Under heat stress, damage to the intestinal mucosal barrier allows a significant influx of harmful substances into the bloodstream that can potentially lead to endotoxemia and a response akin to septic DIC (Lim, 2018). The inflammatory response to heat stress is triggered by inflammasomes and various cytokines that can directly or indirectly induce cytotoxic effects, resulting in cellular apoptosis, necrosis, and pyroptosis (Li et al., 2020). HIC heat stress-induced injury is mediated by host-derived damage-associated molecular patterns (DAMPs), such as pathogen-associated molecular patterns observed in infectious diseases (Geng et al., 2015). These patterns prompt mononuclear macrophages to release inflammatory cytokines such as tumor necrosis factor-alpha (TNF-α), interleukin-1β (IL-1β), and interferon-γ (IFN-γ) through inflammasomes (Zhan et al., 2021). This cytokine release further stimulates the innate immune and coagulation systems and causes reactions such as fever, leukocytosis, and endothelial cell and platelet activation. In addition, activated mononuclear macrophages express tissue factor (TF), which recruits and activates factor VII to form TF-VIIa complexes, directly contributing to thrombus formation via the extrinsic coagulation pathway (Li et al., 2020). Neutrophil extracellular traps (NETs) are promoted by inflammatory factors that stimulate neutrophils. NETs help regulate coagulation factor levels and induce platelet aggregation and activation, attracting components such as vWF, fibrinogen, and fibronectin for thrombus formation. Furthermore, the principal components, nucleosomes and histones, enhance thrombin production, whereas free DNA activates the intrinsic coagulation pathway, thus participating in HIC development (Yao et al., 2023; Fuchs et al., 2010; Ping et al., 2024).

### 3.3 Imbalance between coagulation and fibrinolytic systems

HS increases the expression of TF, which activates factor VII and facilitates thrombin generation via the extrinsic pathway. Concurrently, HS impairs vascular endothelial cells, potentially triggering factor XII through DAMPs, thereby contributing to thrombin production via the intrinsic pathway. This substantial thrombin generation promotes the formation and deposition of cross-linked fibrin clots, leading to numerous microvascular thromboses. Furthermore, thrombin can bind to protease-activated receptor-1 (PAR1) on platelet surfaces and initiate platelet degranulation, leading to the release of vWF and P-selectin, which in turn provoke platelet aggregation and adhesion. These processes culminate in microvascular thrombosis (Ping et al., 2024). A pivotal element in HIC is a disrupted fibrinolytic system, particularly as it progresses to DIC. Tissue-type plasminogen activator (t-PA) and plasminogen activator inhibitor-1 (PAI-1) are two functionally opposing factors that are important for the body to maintain the functional balance of the fibrinolytic system under normal physiological conditions (Ismail et al., 2021). During the early stage of HS, the damaged vascular endothelium releases a large amount of t-PA, and the patient exhibits a state of hyperfibrinolysis. In addition, the patient’s fibrinogen level decreases, leading to hemorrhages (Bouchama et al., 1996). However, during the transition from HIC to DIC, elevated PAI-1 levels inhibit the conversion of plasminogen to plasmin, exceeding the effect of t-PA. This causes a state of fibrinolytic suppression that leads to widespread microthrombus formation. This, in turn, exhausts coagulation factors and other components, causing disruption and eventual breakdown of both the coagulation and fibrinolytic systems (Song et al., 2023).

## 4 HIC treatment

**FIGURE 2:**
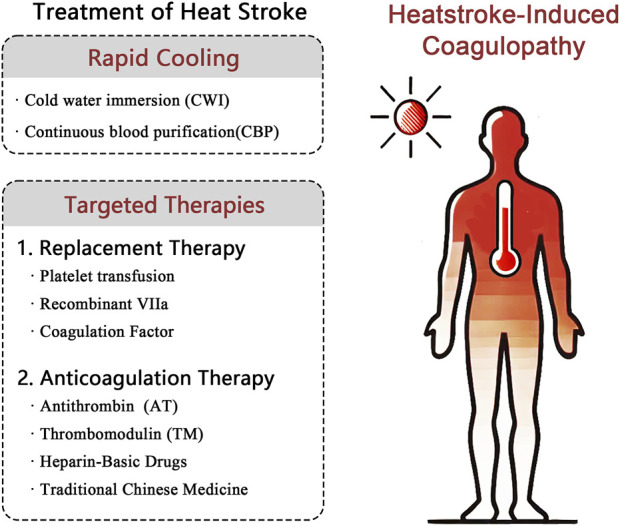
Mechanisms of replacement and anticoagulation therapy for heatstroke-induced coagulopathy. Notes: vWF: von Willebrand factor. APC: Activated protein C.

The core of HS management is rapid cooling and precise management of its fatal complication, HIC. Guidelines require reducing the core body temperature below 39°C within 30 min. Cold water immersion (CWI) and continuous blood purification (CBP) are key cooling methods. HIC manifests as a dynamically evolving DIC-like process that progresses from hypercoagulable tendency (microthrombosis risk) to a consumptive hypocoagulable state (hemorrhage risk). Systematic treatment employs stratified management based on dynamic coagulation monitoring (e.g., thromboelastography) for consumptive hypocoagulability with active bleeding or significant coagulation factor/platelet deficiency (hypofibrinogenemia and platelets <50 × 10^9^/L). It also requires implement replacement therapy (the transfusion of fresh frozen plasma, cryoprecipitate, prothrombin complex concentrate, platelets, and the administration of recombinant factor VIIa if needed). The hypercoagulable phase without active bleeding (monitoring-indicated) requires prompt anticoagulation therapy (antithrombin, thrombomodulin, and heparin-based agents) to inhibit coagulation activation and prevent organ damage. Exploratory approaches represent future directions. HIC management must integrate the “Rapid Cooling→Dynamic Monitoring→Targeted Intervention” strategy (Figure 2).

### 4.1 Heatstroke treatment

The literature suggests that the HS mortality rate is more dependent on the duration of high fever in patients than on the peak temperature reached (Boucha et al., 2022). The primary intervention of HS treatment is the rapid reduction of the patient’s core temperature, with the aim to decrease it to <39°C within the first 30 min to mitigate complications (Expert Group of Heat Stroke, 2023). CWI is the gold standard for exertional heatstroke (EHS), with the aim to achieve a core body temperature cooling rate of 0.35 °C/min (Flouris et al., 2015). Applying large amounts of water to the skin at temperatures between 25°C and 30°C along with enhancing air circulation in elderly patients with chronic HS can result in a cooling rate of 0.10 C/min (Epstein and Yanovich, 2019; Asmara, 2020). CBP serves as a vital intervention for rescuing critically ill patients and is extensively employed in HS treatment (Liu et al., 2020; Zhou et al., 2019). CBP efficiently removes inflammatory mediators and reduces myoglobin and bilirubin accumulation (Zhou et al., 2019; Ikeda et al., 1999; Lumlertgul et al., 2021). Moreover, it alleviates renal and cardiac fluid loads, corrects water, electrolyte, and acid base imbalances, and aids in precise volume management to maintain the internal stability of a patient’s environment (He et al., 2024; Zhou et al., 2011). CBP also enables rapid and safe cooling, making it a crucial technique for effective intravascular cooling in hospitalized patients (Sheng et al., 2021).

### 4.2 HIC alternative treatments

After hemorrhagic shock, widespread bleeding may occur due to the stimulation of mononuclear macrophages through DAMPs. This leads to the release of various cytokines (e.g., TNF-α, IL-6, IL-1β) and TF expression (Iba et al., 2022). This coagulation cascade activation results in the formation of a widespread microthrombus that leads to significant consumption of the body’s coagulation substances. This will ultimately cause consumptive coagulopathy. It is recommended to use coagulation function, thromboelastography, and platelet function analyses for HS-induced hypocoagulopathy as reference indicators for conducting goal-directed coagulation therapy, specifically replacement therapy.

#### 4.2.1 Supplement coagulation factors

Research evidence indicates that heat stress significantly activates the coagulation system and rapidly depletes coagulation factors. This potentially results in massive bleeding (Boucha et al., 2022). Therefore, patients suffering from HS should receive prompt supplementation with coagulation factors, such as fresh frozen plasma (FFP), fibrinogen (Fib), prothrombin complex concentrate (PCC), and cryoprecipitate. When the prothrombin time (PT) or activated partial thromboplastin time (APTT) in a patient’s coagulation indicators extend by greater than 1.5 times, the thromboelastography reaction time (R-value) exceeds 9 min, or the activated clotting time surpasses 160 s (native whole blood) or 240 s (anticoagulated whole blood). The expert consensus recommends prompt administration of an intravenous infusion of FFP (Song et al., 2023). Although evidence suggests that a dose of 30 mL/kg of FFP can more effectively correct coagulation factor levels, the initial dosage is generally recommended to be 15 mL/kg due to consideration of the patient’s volume load (Wada et al., 2014a). The dosage may then be adjusted based on the monitored coagulation indicators. If prolonged PT/APTT does not significantly improve, an additional 200–400 mL of FFP may be administered to address consumptive coagulopathy and restore PT and APTT to normal levels. If the patient’s volume load precludes a large volume of FFP, the PCC should be utilized preferentially to replenish factors II, VII, IX, and X, with the dosage determined by the international normalized ratio (INR) and the patient’s weight (Waters, 2014). If the Fib level is <180 mg/dL, cryoprecipitate (5–10 units per administration) may be administered to correct the deficiency (Epstein and Yanovich, 2019). Although coagulation factor supplementation therapy can alleviate the sudden drop of coagulation factors in patients with HIC to a certain extent, its effect is ultimately limited. As the patient progresses to an advanced stage of HIC, coagulation factors will be depleted too rapidly, and the coagulation factors provided by complementary therapies will not be able to satisfy the body’s supply-demand balance.

#### 4.2.2 Platelet supplementation

A decrease or significant downward trend in platelet count serves as a non-specific yet sensitive DIC marker (Levi et al., 2009). Platelets should be supplemented when the count falls to <50 × 10^9^/L (Squizzato et al., 2016). At such junctures, the transfusion of one unit of apheresis platelets can, in theory, elevate the count by 10–20 × 10^9^/L (Nagrebetsky et al., 2019). Thromboelastography can be used 1 hour after transfusion to dynamically assess platelet-related parameters to determine the necessity and quantity of further platelet transfusions. In cases in which platelets are unavailable or cannot be promptly sourced, alternative therapies, such as recombinant IL-11, recombinant human thrombopoietin, and platelet-enhancing capsules, may be employed to augment the platelet count.

#### 4.2.3 Recombinant coagulation factor VIIa supplementation

If PCC is unavailable and FFP infusion is too time-consuming to promptly reverse life-threatening bleeding, or if effective hemostasis remains unachieved despite active replacement therapy, recombinant human coagulation factor VIIa (rVIIa) may be employed as a rescue therapy (Ageno et al., 2012). Initially it was reported in 1983 for the treatment of bleeding in hemophilia A patients, and the current indications for rVIIa include hemophilia A or B with inhibitors, expected high anamnestic response, acquired hemophilia, congenital factor VII deficiency, and Glanzmann’s thrombasthenia. rVIIa promotes hemostasis via TF-dependent pathways at endothelial injury sites and through platelet-related TF-independent mechanisms (Park, 2021). The rVIIa administration has the following prerequisites: absence of acidosis, hypothermia, and hypocalcemia; hematocrit >24%, platelet count >50 × 10^9^/L, and Fib >1.5 g/L. An initial dose of rVIIa is administered at 100 μg/kg, followed by 50 μg/kg every 2 h, with adjustments made according to the bleeding severity (Liu et al., 2020). Discontinuation timing should be clinician-guided based on the coagulation test results and clinical symptoms. The lack of standardized discontinuation timing resulted in the need for clinicians with relevant experience and expertise when treating HIC patients.

### 4.3 HIC anticoagulation therapy

Severe coagulation disorders frequently occur during HIC progression that necessitate prompt anticoagulation therapy. However, in cases of active bleeding (e.g., intracranial or massive gastrointestinal hemorrhage), it is essential to first address the bleeding before reassessing the timing of anticoagulation therapy. In patients with HIC, the coagulation function is dynamic, and timely intervention can significantly reduce the DIC incidence in patients with HS by providing replacement therapy based on coagulation-related test results while concurrently administering anticoagulation treatments (Wang and Zhang, 2020). Common anticoagulants used in HIC include antithrombin, thrombomodulin, and heparin.

#### 4.3.1 Antithrombin

Antithrombin (AT) is a single-chain glycoprotein produced by the liver that plays a critical role in regulating the coagulation cascade within the blood environment and is considered a key serine protease inhibitor (Bianchini, 2022). AT inhibits factors XIa and VIIa in the extrinsic pathway and factor Xa and thrombin in the common pathway. Furthermore, AT can competitively bind to heparin and significantly reduce its interaction with heparan sulfate on endothelial cells, leading to a 1000-fold increase in AT activity. Thrombin activation of platelets and endothelial cells contributes to increased local inflammation (Sungurlu et al., 2020). AT is recognized for its anti-inflammatory properties, primarily through its antithrombin effects. In Japan, AT has been approved for sepsis-induced DIC treatment (Egi et al., 2021; Wiedermann, 2022). However, its efficacy as a standalone DIC treatment remains unclear (Tanaka et al., 2019). The International Society on Thrombosis and Hemostasis and the Japanese Sepsis Guidelines only weakly endorse the use of AT for DIC when AT activity is diminished. The combined administration of AT and recombinant human thrombomodulin (rhTM) can effectively enhance the platelet count and reduce D-dimer levels in patients with severe sepsis-related DIC and significantly decrease mortality without heightening bleeding risk (Iba et al., 2017; Iba and Thachil, 2016; Yasuda et al., 2016). The concurrent application of AT and rhTM is a viable treatment strategy for clinical DIC management. However, there are no corresponding studies that have reported on the use of AT alone in HIC.

#### 4.3.2 Thrombomodulin (TM)

TM is an endothelial anticoagulant cofactor that is essential for the regulation of intravascular coagulation and the alteration of the role of thrombin from procoagulant to anticoagulant. TM reduces thrombin’s coagulative activity and activates protein C’s anticoagulant properties (Ohbe et al., 2019; Takashi et al., 2014). A multinational, multi-center phase III clinical study on sepsis demonstrated that intravenous rhTM administration significantly decreased the levels of prothrombin fragment 1 + 2 and thrombin–antithrombin complexes, substantially reducing mortality risk (Levi et al., 2020). Matsumoto et al. reported a case of pyrexia DIC that may be worthwhile as a helpful attempt to induce a biphasic transition from hyperfibrinolytic to hypofibrinolytic DIC (Matsumoto et al., 2019). During the initial phase of consumptive coagulopathy and enhanced fibrinolysis, bleeding tendencies were managed with the use of tranexamic acid, FFP, and platelets. During the later phase in which the fibrinolytic phenotype was inhibited, combined anticoagulation therapy that used rhTM-α and antithrombin III concentrate proved to be effective, significantly ameliorating multiple organ failure, including severe liver failure.

#### 4.3.3 Heparin drugs

Heparin is the earliest discovered anticoagulant (Wardrop and Keeling, 2008) and is predominantly sourced from lung, blood vessel wall, and intestinal mucosa tissue. It is extensively used to treat thrombotic diseases. Its mechanism primarily involves binding with antithrombin III (AT-III) to enhance the inhibition of coagulation factors IIa, IXa, Xa, XIa, and XIIa, consequently preventing the conversion of Fib to fibrin and providing an anticoagulant effect (Claudel et al., 2021). Low-molecular-weight heparin (LMWH), a sulfated glycosaminoglycan fragment derived from the enzymatic cleavage of unfractionated heparin (UFH), exhibits a ratio of anti-factor Xa to AT activity of 2–4:1. Compared with UFH, LMWH has a reduced effect on AT, leading to fewer adverse reactions such as heparin-induced bleeding (Shi et al., 2019). Yu et al. (2018) found that early heparin intervention administered 0–1 h after thermal shock EHS-modeled rats improved liver and kidney function, as well as levels of Fib, D-dimer, and platelet counts. However, it further prolonged the APTT. This intervention was also able to significantly improve the 8-h survival rate of modeled rats. Yutang et al. (2015) conducted a prospective randomized controlled trial that compared the effects of LMWH and UFH in patients with EHS during the pre-DIC stage. They found no significant differences in the DIC incidence and mortality between the two groups. Platelet counts and D-dimer levels in patients with HIC were effectively reduced in both treatments. However, LMWH did not significantly prolong APTT compared with UFH, indicating greater safety. In addition, both UFH and LMWH can bind to platelet factor 4, stimulating IgG antibody production that enhances platelet activation and thrombosis, potentially leading to heparin-induced thrombocytopenia (HIT) (Pishko and Cuker, 2017). The prevailing international HIT diagnostic approach involves exclusion and confirmation via the 4Ts score, dynamic monitoring of the platelet count, and HIT antibody detection and/or platelet function tests (Feng et al., 2024). The specific details of the 4Ts scoring are presented in Table 2 (Feng et al., 2024). Heparin should be discontinued and replaced with non-heparin anticoagulants, such as argatroban or bivalirudin (Liu et al., 2020) in highly suspected or confirmed HIT cases.

**TABLE 2:** 4Ts heparin-induced thrombocytopenia score.

Items	Score = 2	Score = 1	Score = 0
Quantitative characteristics of thrombocytopenia	Minimum value ≥ 20 × 10^9^/L and >50% reduction	Minimum value (10–19) × 10^9^/L or 30%–50%	Reduction minimum value < 10 × 10^9^/L or <30% reduction
Time to thrombocytopenia or thrombosis	Using heparin for 5–10 days or having been exposed to heparin in the past 30 days, re-exposure to heparin ≤1 day	Use heparin >10 days or have been exposed to heparin in the past 30–100 days, re-exposure to heparin ≤1 day	No recent exposure to heparin, use of heparin <5 days
Type of thrombosis	Newly formed arteriovenous thrombus or skin necrosis or acute systemic reaction after loading dose of heparin	Progressive or recurrent thrombosis, skin erythema or suspected thrombosis not yet proven	None
Other causes of thrombocytopenia	None	Possibly Yes	Yes

### 4.4 HIC treatment using TCM

TCM has exhibited unique characteristics for HIC prevention and treatment, demonstrating significant therapeutic potential. Basic studies have confirmed that the combination of Gua Sha with bloodletting treatment significantly reduced PT, APTT, and D-dimer levels in patients with HS. This approach also mitigated plasma protein C and platelet count declines, thereby enhancing systemic inflammation control and reducing ischemic damage to multiple organ tissues (Tu et al., 2015). TCM application of a tanshinone IIA sodium sulfonate treatment at a dose of 40 mg/kg was shown by Chen et al. (2017) to effectively reduce the inflammatory response, apoptosis of aortic endothelial cells, and improve coagulation function indexes in a HS rat model. Haematopoietin injection improved abnormal PT, APTT, INR, Fib, and D-dimer indexes in experimental animals before and after HS occurrence (Xu et al., 2015; Ji et al., 2014). Another randomized controlled study confirmed that Chinese rhubarb, as an adjunctive treatment, alleviated the inflammatory response and promoted the recovery of heat-related acute hepatic, renal injury, and coagulation function (Wan et al., 2018).

### 4.5 Emerging HIC treatments

#### 4.5.1 Human umbilical cord blood stem cell (HUCBC) transplantation therapy

HUCBCs are derived from umbilical cord blood, and approximately 2% of HUCBCs exhibit stem cell potential. These cells can differentiate into all cell types of the nervous system, including neurons, oligodendrocytes, and astrocytes (Saporta et al., 2003). HUCBC transplantation therapy involves the HUCBC administration via intravenous injection and targeted brain localization has shown promising therapeutic effects on central nervous system injuries. In recent years, preclinical studies have demonstrated promising results regarding HUCBC use for HS treatment. Preliminary research has revealed that pretreatment with HUCBCs can substantially mitigate arterial hypotension, cerebral ischemia, and hypoxia associated with HS, and reduce inducible nitric oxide synthase (iNOS)-dependent nitric oxide levels in the striatum. This effectively prevents circulatory shock and repairs cerebral ischemic damage, although peripheral blood mononuclear cells show no significant improvement in the pathological outcomes of HS (Chen et al., 2006). Furthermore, both post- and pre-treatment with HUCBCs help to maintain adequate systemic and cerebral circulation by lowering cytokine production and inhibiting iNOS-dependent nitric oxide synthesis in the brain, thus diminishing circulatory shock, brain nitric oxide overload, and ischemic injury, thereby providing protective effects during heat stress (Chen et al., 2007a). In addition, CD34^+^ cells isolated from HUCBCs secrete therapeutic molecules such as glial cell-derived neurotrophic factor that reduce serum TNF expression, alleviate systemic inflammatory responses, and effectively reduce HS-induced hypercoagulability. This significantly lessens plasma PT, APTT, and D-dimer level increases, as well as decreases plasma protein C levels and platelet counts caused by heat stress (Chen et al., 2007b). However, HUCBCs still require further preclinical studies and clinical trials before being marketed. In addition, the high costs and technical problems will be hurdles that may limit its subsequent application.

#### 4.5.2 Targeted NET formulations

Neutrophils are essential to a host’s immune system. They primarily are involved in immune defense through degranulation, phagocytosis, and NET formation. NETs are mesh-like DNA structures adorned with cytoplasmic, granular, and nuclear proteins that neutrophils release to curb pathogen spread or combat larger microorganisms. They play a significant role in various aspects of immune defense, including inflammation, immune and tumor diseases, and thrombosis (Hidalgo et al., 2022; Papayannopoulos, 2018). NETs are predominantly generated via a cell death mechanism known as lytic or suicidal NETosis. They are crucial in the development of deep vein thrombosis by providing a scaffold for platelets, vWF, and fibrin, thereby facilitating the formation of venous thrombi along with TF, coagulation factors, and complement components (Yao et al., 2023). NET components, such as histones H3 and H4, exhibit high cytotoxicity toward endothelial cells and induce platelet aggregation and activation via Toll-like receptors. Activated platelets further augment NET formation, ultimately enhancing thrombin production (Laridan et al., 2017). Previous studies have demonstrated that NETs activate the coagulation cascade. Treatment with DNase I, a common drug used to degrade NET DNA, significantly reduces the thrombin time and D-dimer and TM levels in HS mice. Concurrently, this treatment markedly increases platelet and Fib levels. These results substantiate that NET degradation mitigates DIC risk and enhances survival rates in HS mice (Zhang et al., 2022). In addition, P-selectin, stored in the α-granules of platelets, promotes NET formation by binding to P-selectin glycoprotein ligand-1 (PSGL-1). This interaction is a potential therapeutic target for NET-related diseases. P-selectin/PSGL-1 inhibitors are currently in clinical development, and they diminish inflammation and pathological thrombosis (Etulain et al., 2015). In conclusion, the focus on NETs as a therapeutic target could offer a valuable new HIC treatment strategy.

#### 4.5.3 Activated protein C

Activated Protein C (APC) may be an effective method to address HIC. APC is a physiological anticoagulant that originates from precursor protein C and is capable of inactivating coagulation factors Va and VIIIa. Recent studies have indicated that APC is associated with various clinical conditions, including DIC (Wada et al., 2014b), sepsis (Bech et al., 2018), and ischemic cardiomyopathy (Ren et al., 2019). In addition to its primary anticoagulant function, APC exhibits significant anti-inflammatory, anti-apoptotic, and endothelial barrier protective effects (Xue et al., 2011) that are largely mediated by the cleavage of protease-activated receptor 1 (Oto et al., 2020). Lin et al. (2009) confirmed that recombinant human Activated Protein C (rhAPC) effectively mitigates PT, APTT, and D-dimer level increases. rhAPC also mitigates the decline in platelet count and protein C levels induced by HS. rhAPC has not demonstrated optimal effects in reducing mortality from sepsis, but its anticoagulant and anti-inflammatory properties continue to endorse its potential as a research drug for HIC prevention and treatment. Notably, Eli Lilly’s rhAPC drug, Xigris, previously utilized for treating septic DIC, was withdrawn globally following its inadequate therapeutic efficacy and elevated bleeding risk, as reported in the PROWESS-SHOCK clinical study (Steven et al., 2014). Currently, the Cortellis database lists only Teijin’s CTC-111 that was approved in Japan in 2018 for the indication of DIC. However, other marketed rhAPC drugs that have not been approved for the indication of DIC may hold potential as therapeutic DIC agents (Ping et al., 2024). However, we can only speculate from the therapeutic potential of individual rhAPC products for DIC that they may have therapeutic effects in HIC. Currently, there remains a research gap regarding rhAPC in the field of HIC, and this requires more in-depth studies.

## 5 Summary and outlook

**FIGURE 3:**
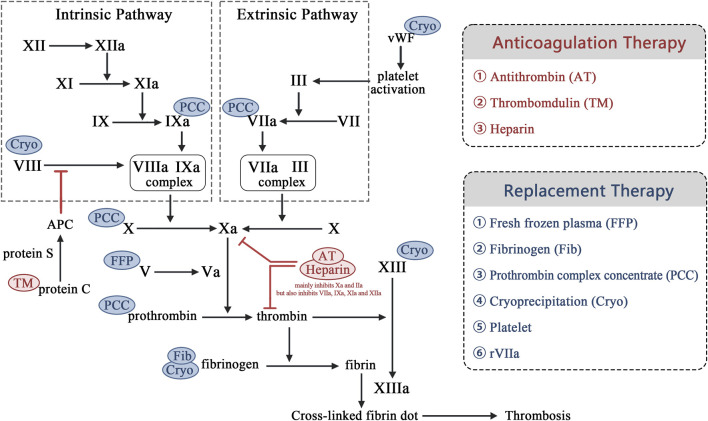
Comprehensive treatment of heatstroke-induced coagulopathy.

There remains an urgent need to identify treatment drugs or methods for HIC that are safer and faster-acting, exhibit fewer side effects, and offer more flexibility and control due to the ambiguous mechanisms, complex conditions, and dynamic shifts of HIC However, no significant advancements have been achieved at this stage. In this review, we summarized our current understanding of HIC pathogenesis and diagnostic guidelines, presented a comprehensive HS treatment strategy, and focused on pharmacological HIC treatment approaches (Figure 3). Alternative therapies, anticoagulant treatments, and TCM interventions have been used to some extent to address DIC induced by HIC or HS, and novel therapies are increasingly capturing the attention of the academic community as research continues to evolve. Furthermore, the recent introduction of new oral anticoagulants has broadened the therapeutic options for coagulation system disorders. Additionally, the development of novel factor XIa inhibitors promises to enhance the precision and safety of anticoagulation strategies. These approaches also have the potential to be applied in HIC emergency interventions. However, the current limitations of HIC treatment remain intractable, and there are some therapeutic controversies, especially regarding emerging therapies with limited clinical validation. Therapeutic regimens for HS such as CWI and CBP show promise for HIC prevention, but few studies have explored HIC replacement therapy (e.g., FFP, Fib, PCC, cryoprecipitate, platelets, and recombinant coagulation factor VIIa) and HIC anticoagulation therapy (e.g., antithrombin, thrombomodulin, and heparin) remain controversial in terms of application timing. These treatments need to be administered by experienced clinicians. TCM has been involved in HIC treatment, but it often lacks the support of molecular biological mechanism research and high-level clinical trial evidence based on modern research techniques. Only a few preclinical studies have been conducted on HUCBC for HS treatment, and they have primarily focused on blood circulation and inflammation effects and less on the protection of the coagulation system (Chen et al., 2007b). NETs target-related P-selectin/PSGL-1 inhibitors are still under development, and there are no clinically approved therapeutic drugs available (Etulain et al., 2015). rhAPC has mechanistically been able to effectively exert an anticoagulant effect. But it has shown poor therapeutic efficacy and high therapeutic risk in septic DIC treatment, which is similar to HIC. This duality has polarized scientific expectations for its clinical application. Therefore, there is no perfect therapeutic solution or drug, and further elucidation of the pathological mechanisms of HIC development based on the existing research is required in order to search for a higher level of evidence-based medicine to support more effective and specific HIC treatments.

## References

[B1] Ageno W. Gallus A. S. Wittkowsky A. Crowther M. Hylek E. M. Palareti G. (2012). Oral anticoagulant therapy: Antithrombotic therapy and prevention of thrombosis, 9th ed: American college of chest physicians evidence-based clinical practice guidelines. Chest 141 (2 Suppl. l), e44S-e88S–e88S. 10.1378/chest.11-2292 PMC3278051 22315269

[B2] Asmara I. (2020). Diagnosis and management of heatstroke. Acta Med. Indones. 52 (1), 90–97. 32291378

[B3] Becher T. Müller J. Akin I. Baumann S. Bosch K. Stach K. (2018). The evolution of activated protein C plasma levels in septic shock and its association with mortality: a prospective observational study. J. Crit. Care 47, 41–48. 10.1016/j.jcrc.2018.06.003 29886066

[B4] Bianchini E. P. (2022). Antithrombin deficiency: no sugar, no diagnosis. Blood 140 (2), 83–85. 10.1182/blood.2022016677 35834281

[B5] Bouchama A. Abuyassin B. Lehe C. Laitano O. Jay O. O'Connor F. G. (2022). Classic and exertional heatstroke. Nat. Rev. Dis. Prim. 8 (1), 8. 10.1038/s41572-021-00334-6 35115565

[B6] Bouchama A. Bridey F. Hammami M. M. Lacombe C. al-Shail E. al-Ohali Y. (1996). Activation of coagulation and fibrinolysis in heatstroke. Thromb. Haemost. 76 (6), 0909–0915. 10.1055/s-0038-1650685 8972010

[B7] Chen F. Li H. Zhu G. Chen X. Tang Z. (2017). Sodium tanshinone IIA sulfonate improves inflammation, aortic endothelial cell apoptosis, disseminated intravascular coagulation and multiple organ damage in a rat heat stroke model. Mol. Med. Rep. 16 (1), 87–94. 10.3892/mmr.2017.6573 28498471 PMC5482147

[B8] Chen S. H. Chang F. M. Chang H. K. Chen W. C. Huang K. F. Lin M. T. (2007b). Human umbilical cord blood-derived CD34+ cells cause attenuation of multiorgan dysfunction during experimental heatstroke. Shock (Augusta, Ga.) 27 (6), 663–671. 10.1097/01.shk.0000248593.71388.40 17505307

[B9] Chen S. H. Chang F. M. Tsai Y. C. Huang K. F. Lin C. L. Lin M. T. (2006). Infusion of human umbilical cord blood cells protect against cerebral ischemia and damage during heatstroke in the rat. Exp. Neurol. 199 (1), 67–76. 10.1016/j.expneurol.2005.11.015 16405889

[B10] Chen S. H. Huang K. F. Lin M. T. Chang F. M. (2007a). Human umbilical cord blood cells or estrogen May be beneficial in treating heatstroke. Taiwan J. Obstet. Gynecol. 46 (1), 15–25. 10.1016/S1028-4559(08)60101-1 17389184

[B11] Claudel S. E. Miles L. A. Murea M. (2021). Anticoagulation in hemodialysis: a narrative review. Seminars Dialysis. 34 (2), 103–115. 10.1111/sdi.12932 33135208

[B12] Egi M. Ogura H. Yatabe T. Atagi K. Inoue S. Iba T. (2021). The Japanese clinical practice guidelines for management of sepsis and septic shock 2020 (J-SSCG 2020). J. Intensive Care 9 (1), 53. 10.1186/s40560-021-00555-7 34433491 PMC8384927

[B13] Epstein Y. Yanovich R. (2019). Heatstroke. N. Engl. J. Med. 380 (25), 2449–2459. 10.1056/NEJMra1810762 31216400

[B14] Etulain J. Martinod K. Wong S. L. Cifuni S. M. Schattner M. Wagner D. D. (2015). P-selectin promotes neutrophil extracellular trap formation in mice. Blood 126 (2), 242–246. 10.1182/blood-2015-01-624023 25979951 PMC4497964

[B15] Expert group of heat stroke prevention and treatment of the Chinese PLA. Expert consensus on cooling methods for prevention heat Illness/stroke in military training. Med. J. Chin. PLA (2023). 48 (8): 871–878. 10.11855/j.issn.0577-7402.1888.2023.0105

[B16] Feng L. Yin J. Y. Liu Y. H. Zhang P. Zhao Y. L. Song Q. (2024). N-terminal pro-brain natriuretic peptide - a significant biomarker of disease development and adverse prognosis in patients with exertional heat stroke. Mil. Med. Res. 11 (1), 26. 10.1186/s40779-024-00531-w 38654334 PMC11036771

[B17] Flouris A. D. Friesen B. J. Carlson M. J. Casa D. J. Kenny G. P. (2015). Effectiveness of cold water immersion for treating exertional heat stress when immediate response is not possible. Scand. J. Med. Sci. Sports 25 (Suppl. 1), 229–239. 10.1111/sms.12317 25943674

[B18] Fuchs T. A. Brill A. Duerschmied D. Schatzberg D. Monestier M. Myers D. D. (2010). Extracellular DNA traps promote thrombosis. Proc. Natl. Acad. Sci. U. S. A. 107 (36), 15880–15885. 10.1073/pnas.1005743107 20798043 PMC2936604

[B19] Geng Y. Ma Q. Liu Y. N. Peng N. Yuan F. F. Li X. G. (2015). Heatstroke induces liver injury *via* IL-1β and HMGB1-induced pyroptosis. J. Hepatol. 63 (3), 622–633. 10.1016/j.jhep.2015.04.010 25931416

[B20] Han Q. Liu Z. Jia J. Anderson B. T. Xu W. Shi P. (2022). Web-based data to quantify meteorological and geographical effects on heat stroke: case study in China. Geohealth 6 (8), e2022GH000587. 10.1029/2022GH000587 PMC9356531 35949256

[B21] He Y. Cheng Y. Chen M. (2024). Clinical application of continuous blood purification in sepsis complicated with acute respiratory distress syndrome. Minerva Surg. 79 (4), 487–489. 10.23736/S2724-5691.21.09228-5 34672488

[B22] Hidalgo A. Libby P. Soehnlein O. Aramburu I. V. Papayannopoulos V. Silvestre-Roig C. (2022). Neutrophil extracellular traps: from physiology to pathology. Cardiovasc Res. 118 (13), 2737–2753. 10.1093/cvr/cvab329 34648022 PMC9586562

[B23] Hifumi T. Kondo Y. Shimazaki J. Oda Y. Shiraishi S. Wakasugi M. (2018). Prognostic significance of disseminated intravascular coagulation in patients with heat stroke in a nationwide registry. J. Crit. Care 44, 306–311. 10.1016/j.jcrc.2017.12.003 29253838

[B24] Iba T. Connors J. M. Levi M. Levy J. H. (2022). Heatstroke-induced coagulopathy: biomarkers, mechanistic insights, and patient management. EClinicalMedicine 44, 101276. 10.1016/j.eclinm.2022.101276 35128366 PMC8792067

[B25] Iba T. Hagiwara A. Saitoh D. Anan H. Ueki Y. Sato K. (2017). Effects of combination therapy using antithrombin and thrombomodulin for sepsis-associated disseminated intravascular coagulation. Ann. Intensive Care 7 (1), 110. 10.1186/s13613-017-0332-z 29098447 PMC5668219

[B26] Iba T. Helms J. Connors J. M. Levy J. H. (2023b). The pathophysiology, diagnosis, and management of sepsis-associated disseminated intravascular coagulation. J. Intensive Care 11 (1), 24. 10.1186/s40560-023-00672-5 37221630 PMC10202753

[B27] Iba T. Helms J. Levi M. Levy J. H. (2023a). Inflammation, coagulation, and cellular injury in heat-induced shock. Inflamm. Res. 72 (3), 463–473. 10.1007/s00011-022-01687-8 36609608

[B28] Iba T. Helms J. Levi M. Levy J. H. (2023c). The role of platelets in heat-related illness and heat-induced coagulopathy. Thromb. Res. 231, 152–158. 10.1016/j.thromres.2022.08.009 35989192

[B29] Iba T. Thachil J. (2016). Present and future of anticoagulant therapy using antithrombin and thrombomodulin for sepsis-associated disseminated intravascular coagulation: a perspective from Japan. Int. J. Hematol. 103 (3), 253–261. 10.1007/s12185-015-1904-z 26588929

[B30] Ikeda Y. Sakemi T. Nishihara G. Nakamura M. Fujisaki T. Koh T. (1999). Efficacy of blood purification therapy for heat stroke presenting rapid progress of multiple organ dysfunction syndrome: a comparison of five cases. Intensive Care Med. 25 (3), 315–318. 10.1007/s001340050842 10229168

[B31] Ismail A. A. Shaker B. T. Bajou K. (2021). The plasminogen-activator plasmin system in physiological and pathophysiological angiogenesis. Int. J. Mol. Sci. 23 (1), 337. 10.3390/ijms23010337 35008762 PMC8745544

[B32] Ji J. Zhou F. Yue H. Song Q. (2014). Protective mechanism of xuebijing injection against heat stroke in rats. Exp. Ther. Med. 7 (6), 1745–1751. 10.3892/etm.2014.1639 24926378 PMC4043590

[B33] Khan A. Mubeen M. (2025). Heat stroke in the era of global warming: a call for urgent action. Ann. Glob. Health 91 (1), 1. 10.5334/aogh.4519 39867166 PMC11760228

[B34] Laridan E. Denorme F. Desender L. François O. Andersson T. Deckmyn H. (2017). Neutrophil extracellular traps in ischemic stroke thrombi. Ann. Neurol. 82 (2), 223–232. 10.1002/ana.24993 28696508

[B35] Levi M. Toh C. H. Thachil J. Watson H. G. (2009). Guidelines for the diagnosis and management of disseminated intravascular coagulation. British committee for standards in haematology. Br. J. Haematol. 145 (1), 24–33. 10.1111/j.1365-2141.2009.07600.x 19222477

[B36] Levi M. Vincent J. L. Tanaka K. Radford A. H. Kayanoki T. Fineberg D. A. (2020). Effect of a recombinant human soluble thrombomodulin on baseline coagulation biomarker levels and mortality outcome in patients with sepsis-associated coagulopathy. Crit. Care Med. 48 (8), 1140–1147. 10.1097/CCM.0000000000004426 32697484 PMC7365672

[B37] Li Y. Zhu X. Wang G. Tong H. Su L. Li X. (2020). Proteomic analysis of extracellular vesicles released from heat-stroked hepatocytes reveals promotion of programmed cell death pathway. Biomed. and Pharmacother. 129, 110489. 10.1016/j.biopha.2020.110489 32768969

[B38] Lim C. L. (2018). Heat sepsis precedes heat toxicity in the pathophysiology of heat Stroke-A new paradigm on an ancient disease. Antioxidants (Basel) 7 (11), 149. 10.3390/antiox7110149 30366410 PMC6262330

[B39] Lin Q. W. Zhong L. C. He L. P. Zeng Q. B. Zhang W. Song Q. (2023). A newly proposed heatstroke-induced coagulopathy score in patients with heat illness: a multicenter retrospective study in China. Chin. J. Traumatol. 27 (2), 83–90. 10.1016/j.cjtee.2023.08.001 37625936 PMC11075100

[B40] Lin X. J. Li Y. L. Mei G. P. Zou F. He D. D. Liu X. Q. (2009). Activated protein C can be used as a prophylactic as well as a therapeutic agent for heat stroke in rodents. Shock (Augusta, Ga.) 32 (5), 524–529. 10.1097/SHK.0b013e3181a1a75d 19295493

[B41] Liu S. Y. Song J. C. Mao H. D. Zhao J. B. Song Q. (2020). Expert consensus on the diagnosis and treatment of heat stroke in China. Mil. Med. Res. 7 (1), 1. 10.1186/s40779-019-0229-2 31928528 PMC6956553

[B42] Lumlertgul N. Hall A. Camporota L. Crichton S. Ostermann M. (2021). Clearance of inflammatory cytokines in patients with septic acute kidney injury during renal replacement therapy using the EMiC2 filter (Clic-AKI study). Crit. Care 25 (1), 39. 10.1186/s13054-021-03476-x 33509215 PMC7845048

[B43] Majeed E. N. (2009). Effects of heat on camel platelet structure and function--a comparative study with humans. Platelets 20 (7), 528. 10.3109/09537100903207513 19852694

[B44] Matsumoto H. Takeba J. Umakoshi K. Nakabayashi Y. Moriyama N. Annen S. (2019). Successful treatment for disseminated intravascular coagulation (DIC) corresponding to phenotype changes in a heat stroke patient. J. Intensive Care 7, 2. 10.1186/s40560-019-0359-3 PMC6332900 30675362

[B45] Misset B. De Jonghe B. Bastuji-Garin S. Gattolliat O. Boughrara E. Annane D. (2006). Mortality of patients with heatstroke admitted to intensive care units during the 2003 heat wave in France: a national multiple-center risk-factor study. Crit. Care Med. 34 (4), 1087–1092. 10.1097/01.CCM.0000206469.33615.02 16484920

[B46] Nagrebetsky A. Al-Samkari H. Davis N. M. Kuter D. J. Wiener-Kronish J. P. (2019). Perioperative thrombocytopenia: evidence, evaluation, and emerging therapies. Br. J. Anaesth. 122 (1), 19–31. 10.1016/j.bja.2018.09.010 30579402

[B47] Ohbe H. Isogai S. Jo T. Matsui H. Fushimi K. Yasunaga H. (2019). Treatment with antithrombin or thrombomodulin and mortality from heatstroke-induced disseminated intravascular coagulation: a nationwide observational study. Semin. Thromb. Hemost. 45 (8), 760–766. 10.1055/s-0039-1700520 31627216

[B48] Oto J. Fernández-Pardo Á. Miralles M. Plana E. España F. Navarro S. (2020). Activated protein C assays: a review. Clin. Chim. Acta 502, 227–232. 10.1016/j.cca.2019.11.005 31730817

[B49] Papayannopoulos V. (2018). Neutrophil extracellular traps in immunity and disease. Nat. Rev. Immunol. 18 (2), 134–147. 10.1038/nri.2017.105 28990587

[B50] Park J. A. (2021). Treatment of diffuse alveolar hemorrhage: controlling inflammation and obtaining rapid and effective hemostasis. Int. J. Mol. Sci. 22 (2), 793. 10.3390/ijms22020793 33466873 PMC7830514

[B51] Ping P. Yang T. Ning C. Zhao Q. Zhao Y. Yang T. (2024). Chlorogenic acid attenuates cardiac hypertrophy *via* up-regulating Sphingosine-1-phosphate receptor1 to inhibit endoplasmic reticulum stress. Esc. Heart Fail 11 (3), 1580–1593. 10.1002/ehf2.14707 38369950 PMC11098655

[B52] Pishko A. M. Cuker A. (2017). Heparin-induced thrombocytopenia in cardiac surgery patients. Semin. Thromb. Hemost. 43 (7), 691–698. 10.1055/s-0037-1602664 28597462 PMC6022744

[B53] Ren D. Giri H. Li J. Rezaie A. R. (2019). The cardioprotective signaling activity of activated protein C in heart failure and ischemic heart diseases. Int. J. Mol. Sci. 20 (7), 1762. 10.3390/ijms20071762 30974752 PMC6479968

[B54] Saporta S. Kim J. J. Willing A. E. Fu E. S. Davis C. D. Sanberg P. R. (2003). Human umbilical cord blood stem cells infusion in spinal cord injury: engraftment and beneficial influence on behavior. J. Hematother Stem Cell Res. 12 (3), 271–278. 10.1089/152581603322023007 12857368

[B55] Sheng C. Yang C. Jing Z. Li Y. (2021). The use of continuous blood purification for the treatment of malignant hyperthermia in an infant. J. Cardiothorac. Vasc. Anesth. 35 (11), 3307–3310. 10.1053/j.jvca.2020.10.055 33223381

[B56] Shi D. Yao Y. Cheng X. Hui X. Shan Y. Jing Y. (2019). LMWH and its derivatives represent new rational for cancer therapy: construction strategies and combination therapy. Drug Discov. Today 24 (10), 2096–2104. 10.1016/j.drudis.2019.06.011 31228613

[B57] Song J. C. Song Q. Zhang W. Liu S. Y. Li W. Q. Zhou Z. (2023). Expert consensus on the diagnosis and treatment of heat stroke-induced coagulopathy in China. Med. J. Chin. PLA 48 (11), 1237–1247. 10.11855/j.issn.0577-7402.1015.2023.0920

[B58] Song J. C. Song Q. Zhang W. Li W. Q. Zhang X. J. Liu S. Y. (2025). Chinese guidelines for the diagnosis and treatment of heat stroke (2025 edition). Med. J. Chin. PLA 50 (4), 367–386. 10.11855/j.issn.0577-7402.0506.2025.0328

[B59] Squizzato A. Hunt B. J. Kinasewitz G. T. Wada H. Ten Cate H. Thachil J. (2016). Supportive management strategies for disseminated intravascular coagulation. An international consensus. Thromb. Haemost. 115 (5), 896–904. 10.1160/TH15-09-0740 26676927

[B60] Steven M. O. R Phillip D. Jean-Louis V. Henry M. Derek C. A. (2014). The next generation of sepsis clinical trial designs: what is next after the demise of recombinant human activated protein C? Crit. Care Med. 42 (7), 1714–1721. 10.1097/CCM.0000000000000325 24717456 PMC4135726

[B61] Sungurlu S. Kuppy J. Balk R. A. (2020). Role of antithrombin III and tissue factor pathway in the pathogenesis of sepsis. Crit. Care Clin. 36 (2), 255–265. 10.1016/j.ccc.2019.12.002 32172812

[B62] Takashi K. Kohji O. Chika K. Takeyoshi S. (2014). Thrombomodulin improved liver injury, coagulopathy, and mortality in an experimental heatstroke model in mice. Anesth. Analg. 118 (5), 956–963. 10.1213/ANE.0000000000000170 24781566

[B63] Tanaka K. Takeba J. Matsumoto H. Ohshita M. Annen S. Moriyama N. (2019). Anticoagulation therapy using rh-Thrombomodulin And/Or antithrombin III agent is associated with reduction in in-Hospital mortality in septic disseminated intravascular coagulation: a nationwide registry study. Shock 51 (6), 713–717. 10.1097/SHK.0000000000001230 31090683

[B64] Tu W. Z. Cheng R. D. Hu J. Wang J. z. Lin H. y. Zou E. m. (2015). Combination treatment with gua sha and blood-letting causes attenuation of systemic inflammation, activated coagulation, tissue ischemia and injury during heatstroke in rats. Chin. J. Integr. Med. 21 (8), 610–617. 10.1007/s11655-014-1816-4 25098257

[B65] Umemura Y. Ogura H. Matsuura H. Ebihara T. Shimizu K. Shimazu T. (2018). Bone marrow-derived mononuclear cell therapy can attenuate systemic inflammation in rat heatstroke. Scand. J. Trauma Resusc. Emerg. Med. 26 (1), 97. 10.1186/s13049-018-0566-2 30445981 PMC6240199

[B66] Wada H. Matsumoto T. Yamashita Y. (2014a). Diagnosis and treatment of disseminated intravascular coagulation (DIC) according to four DIC guidelines. J. Intensive Care 2 (1), 15. 10.1186/2052-0492-2-15 25520831 PMC4267589

[B67] Wada H. Matsumoto T. Yamashita Y. Hatada T. (2014b). Disseminated intravascular coagulation: testing and diagnosis. Clin. Chim. Acta 436, 130–134. 10.1016/j.cca.2014.04.020 24792730

[B68] Wan Y. Sun S. S. Fu H. Y. Xu Y. K. Liu Q. Yin J. T. (2018). Adjuvant rhubarb alleviates organs dysfunction and inhibits inflammation in heat stroke. Exp. Ther. Med. 16 (2), 1493–1498. 10.3892/etm.2018.6327 30116399 PMC6090376

[B69] Wang J. Zhang Y. (2020). Experience of treating batches of exertional heat stroke patients in military training. Zhonghua wei zhong bing ji jiu yi xue 32 (12), 1522–1525. 10.3760/cma.j.cn121430-20200303-00236 33541509

[B70] Wardrop D. Keeling D. (2008). The story of the discovery of heparin and warfarin. Br. J. Haematol. 141 (6), 757–763. 10.1111/j.1365-2141.2008.07119.x 18355382

[B71] Waters J. H. (2014). Role of the massive transfusion protocol in the management of haemorrhagic shock. Br. J. Anaesth. 113 (Suppl. 2), ii3–ii8. 10.1093/bja/aeu379 25498580

[B72] Wiedermann C. J. (2022). Antithrombin as therapeutic intervention against sepsis-induced coagulopathy and disseminated intravascular coagulation: lessons learned from COVID-19-Associated coagulopathy. Int. J. Mol. Sci. 23 (20), 12474. 10.3390/ijms232012474 36293332 PMC9604230

[B73] Xu Q. Liu J. Guo X. Tang Y. Zhou G. Liu Y. (2015). Xuebijing injection reduces organ injuries and improves survival by attenuating inflammatory responses and endothelial injury in heatstroke mice. BMC Complement. Altern. Med. 15, 4. 10.1186/s12906-015-0519-5 25653103 PMC4323134

[B74] Xue M. Chow S. O. Dervish S. Chan Y. K. Julovi S. M. Jackson C. J. (2011). Activated protein C enhances human keratinocyte barrier integrity *via* sequential activation of epidermal growth factor receptor and Tie2. J. Biol. Chem. 286 (8), 6742–6750. 10.1074/jbc.M110.181388 21173154 PMC3057790

[B75] Yamazawa T. Kobayashi T. Kurebayashi N. Konishi M. Noguchi S. Inoue T. (2021). A novel RyR1-selective inhibitor prevents and rescues sudden death in mouse models of malignant hyperthermia and heat stroke. Nat. Commun. 12 (1), 4293. 10.1038/s41467-021-24644-1 34257294 PMC8277899

[B76] Yao M. Ma J. Wu D. Fang C. Wang Z. Guo T. (2023). Neutrophil extracellular traps mediate deep vein thrombosis: from mechanism to therapy. Front. Immunol. 14, 1198952. 10.3389/fimmu.2023.1198952 37680629 PMC10482110

[B77] Yasuda N. Goto K. Ohchi Y. Abe T. Koga H. Kitano T. (2016). The efficacy and safety of antithrombin and recombinant human thrombomodulin combination therapy in patients with severe sepsis and disseminated intravascular coagulation. J. Crit. Care 36, 29–34. 10.1016/j.jcrc.2016.06.008 27546744

[B78] Yu Y. Wei Y. Zhang X. Li X. (2018). Effect of early intervention with heparin on function of coagulopathy, liver and kidney in rats with exertional heatstroke under the ambient air of high temperature and low humidity. Zhonghua wei zhong bing ji jiu yi xue 30 (3), 214–219. 10.3760/cma.j.issn.2095-4352.2018.03.005 29519278

[B79] Yutang L. Chunwen G. Hui L. Yuan Z. Lin H. Wang Y. (2015). Comparison of the effect of low molecular weight heparin sodium and that of heparin sodium on pre-disseminated intravascular coagulation stage in patients suffering from exertional heat stroke. Zhonghua Wei Zhong Bing Ji Jiu Yi Xue 27 (8), 649–652. 10.3760/cma.j.issn.2095-4352.2015.08.006 26255012

[B80] Zhang Y. Deng X. Zhang J. Zhang L. Akram Z. Zhang B. (2022). A potential driver of disseminated intravascular coagulation in heat stroke mice: neutrophil extracellular traps. Int. J. Environ. Res. Public Health 19 (19), 12448. 10.3390/ijerph191912448 36231751 PMC9566744

[B81] Zhang Y. Khalid S. Jiang L. (2019). Diagnostic and predictive performance of biomarkers in patients with sepsis in an intensive care unit. J. Int. Med. Res. 47 (1), 44–58. 10.1177/0300060518793791 30477377 PMC6384460

[B82] Zhang Z. T. Gu X. L. Zhao X. He X. Shi H. W. Zhang K. (2021). NLRP3 ablation enhances tolerance in heat stroke pathology by inhibiting IL-1β-mediated neuroinflammation. J. Neuroinflammation 18 (1), 128. 10.1186/s12974-021-02179-y 34092247 PMC8182902

[B83] Zhong L. Wu M. Ji J. Liu Z. (2022). Usefulness of sequential organ failure assessment score on admission to predict the 90-day mortality in patients with exertional heatstroke: an over 10-year intensive care survey. Am. J. Emerg. Med. 61, 56–60. 10.1016/j.ajem.2022.08.042 36049393

[B84] Zhou F. Song Q. Peng Z. Pan L. Kang H. Tang S. (2011). Effects of continuous venous-venous hemofiltration on heat stroke patients: a retrospective study. J. Trauma 71 (6), 1562–1568. 10.1097/TA.0b013e31822a71c2 22182867

[B85] Zhou R. X. Dai W. Hu C. L. (2019). Differential clinical benefits of continuous blood purification treatment in critically ill patients with variable APACHE II scores. Exp. Ther. Med. 18 (1), 741–746. 10.3892/etm.2019.7617 31281452 PMC6580099

